# VaccineDA: Prediction, design and genome-wide screening of oligodeoxynucleotide-based vaccine adjuvants

**DOI:** 10.1038/srep12478

**Published:** 2015-07-27

**Authors:** Gandharva Nagpal, Sudheer Gupta, Kumardeep Chaudhary, Sandeep Kumar Dhanda, Satya Prakash, Gajendra P. S. Raghava

**Affiliations:** 1 Bioinformatics Centre, CSIR-Institute of Microbial Technology, Chandigarh-160036, INDIA

## Abstract

Immunomodulatory oligodeoxynucleotides (IMODNs) are the short DNA sequences that activate the innate immune system via toll-like receptor 9. These sequences predominantly contain unmethylated CpG motifs. In this work, we describe VaccineDA (Vaccine DNA adjuvants), a web-based resource developed to design IMODN-based vaccine adjuvants. We collected and analyzed 2193 experimentally validated IMODNs obtained from the literature. Certain types of nucleotides (e.g., T, GT, TC, TT, CGT, TCG, TTT) are dominant in IMODNs. Based on these observations, we developed support vector machine-based models to predict IMODNs using various compositions. The developed models achieved the maximum Matthews Correlation Coefficient (MCC) of 0.75 with an accuracy of 87.57% using the pentanucleotide composition. The integration of motif information further improved the performance of our model from the MCC of 0.75 to 0.77. Similarly, models were developed to predict palindromic IMODNs and attained a maximum MCC of 0.84 with the accuracy of 91.94%. These models were evaluated using a five-fold cross-validation technique as well as validated on an independent dataset. The models developed in this study were integrated into VaccineDA to provide a wide range of services that facilitate the design of DNA-based vaccine adjuvants (http://crdd.osdd.net/raghava/vaccineda/).

In the post-genomic era, where thousands of genomes have already been sequenced including the human genome, it is important to develop computer-aided techniques to design vaccines. Previously, numerous *in silico* tools have been developed to predict antigenic regions or epitopes that can activate the adaptive immune syst*e*m, including prediction of B-cell^1^,^2^,^3^,^4^,^5^,^6^,^7^,^8^,^9^,^10^ and T-cell epitopes^11^,^12^,^13^,^14^,^15^,^16^,^17^,^18^. The scientific community, engaged in designing subunit vaccines, heavily uses these epitope prediction tools. In contrast, there have been a limited number of attempts towards the development of computational resources to develop adjuvants that can activate the innate immune system. Therefore, there is a need to develop *in silico* tools to design adjuvants that can evoke the innate immune responses.

The cells of innate immunity recognize disease-causing pathogens using receptors called pathogen/pattern recognition receptors (PRRs). Previously, our group developed the Pattern-Recognition Receptors Database (PRRDB) that compiles pattern recognition receptors and their ligands^19^. The ligands, which are recognized by PRRs, may serve as vaccine adjuvants because these ligands have the ability to stimulate the innate immune system^20^. One of the important PRRs is toll-like receptor 9 (TLR9) that explicitly recognizes DNA-oligodeoxynucleotides, primarily non-methylated cytosine-phosphate-guanine (CpG) dinucleotides^21^. These oligodexoynucleotides may serve as potential vaccine adjuvants as they can activate the immune system through stimulation of innate immunity^22^. Thus, it is important to understand the characteristics of the immunomodulatory oligodexoynucleotides to design efficient adjuvants. For the sake of simplicity, the term “oligodeoxynucleotide” is referred to as “oligonucleotide” in this work.

In the present work, we described a web-based resource built to aid in the design of oligonucleotides (ODNs)-based adjuvants. ODNs that stimulate the innate immunity are referred to as immunomodulatory oligonucleotides (IMODNs). Similarly ODNs that cannot activate the immune system are referred to as non-IMODNs. First, we collected experimentally well-characterized IMODNs from the literature, mainly from patents. We also compiled palindromic oligonucleotides that may activate the immune system; referred to as palindromic IMODNs. These IMODNs were analyzed to identify nucleotides and motifs that are more prominent in IMODNs. Based on our analysis, we identified features that can be used to discriminate between IMODNs and non-IMODNs. Using these features, we developed models to predict IMODNs with high precision. Finally, we developed a web-based platform/resource referred to as VaccineDA (“Vaccine DNA Adjuvants”), which is freely available to the community at http://crdd.osdd.net/raghava/vaccineda/.

## Results

### Compositional analysis of IMODNs

#### Mononucleotide composition [MNC]

**Figure 1:**
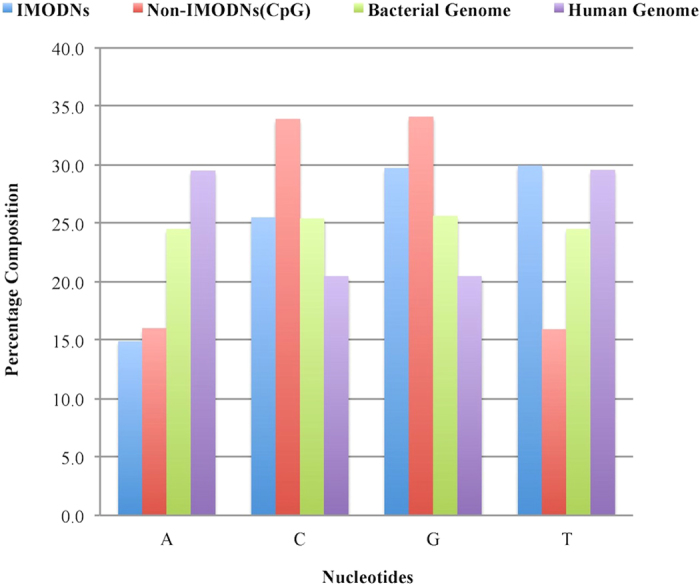
Bar graph showing average percent nucleotide composition of IMODNs, non-IMODNs, bacterial genomes and the human genome.

We computed and compared the nucleotide compositions of IMODN and non-IMODN sequences in the realistic dataset ‘IMODN2193R_train’ (Fig. 1 and Table S1). The dataset IMODN2193R_train contained 1754 experimentally validated IMODNs and 17544 non-IMODNs (randomly selected human CpG island fragments). In addition, we also computed the nucleotide composition of the human genome and 11 bacterial genomes. As illustrated in Fig. 1, the composition of all four nucleotides was nearly the same at ~25% in the case of bacterial genomes. It was observed that the percent composition of thymine (T) in IMODNs (~30%) is significantly higher than that in non-IMODNs (~16%). Similarly, cytosine (C) is less frequent in IMODNs (~25%) than in non-IMODNs (~34%). The composition of adenine (A) is nearly the same in both IMODNs and non-IMODNs; i.e., the difference in the compositions is not significant. The composition of guanine (G) is slightly higher in the case of non-IMODNs in comparison to IMODNs.

#### Dinucleotide composition [DNC]

**Figure 2:**
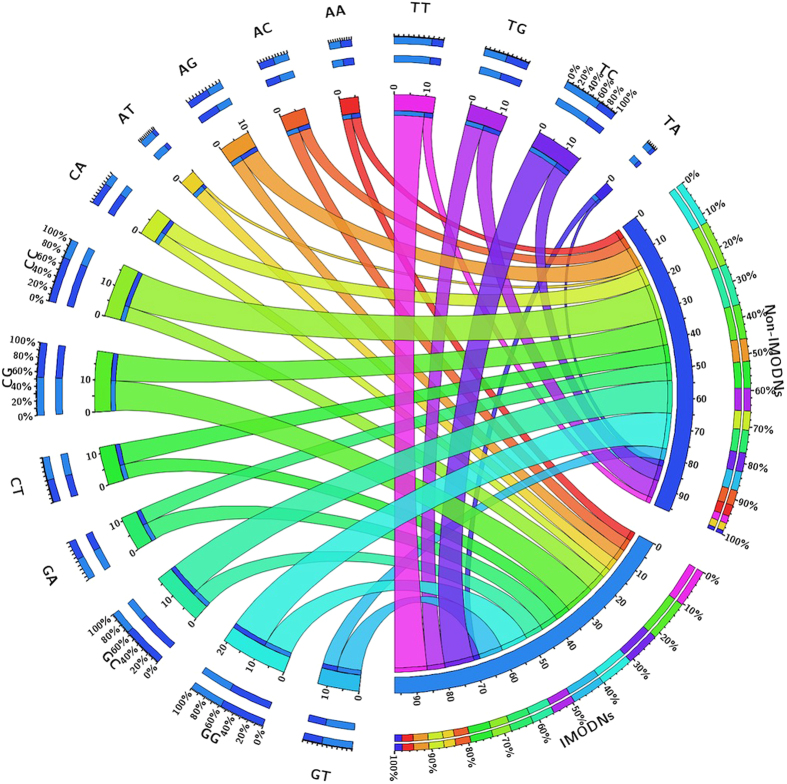
Dinucleotide composition of IMODNs and non-IMODNs represented by Circos Plot. The width of the ribbons shows average percent composition of the dinucleotides in IMODNs and non-IMODNs.

The dinucleotide composition of IMODN and non-IMODN sequences in dataset IMODN2193R_train is shown in Fig. 2 and Table S2. Almost all the dinucleotides containing T are more abundant in IMODNs than non-IMODNs. As shown in Table S2 as well as in Fig. 2, dinucleotides TT, TC, GT, and AT are more prevalent in IMODNs. In contrast, frequencies of dinucleotides like CC, GC, GG and AG are very poor in IMODNs. No significant compositional differences were observed for certain types of dinucleotides like CG, TA, TG, AA, GA, AC, CT, CA.

#### Trinucleotide composition [TNC]

We investigated the contribution of different types of trinucleotides in IMODNs; trinucleotides provide more information as they represent a local order in the sequences. It has been observed that trinucleotides like TTT, CGT, TCG and GTT, are more frequent in IMODNs (Fig. S1 and Table S3). In contrast, certain types of trinucleotides (e.g., GGC, GCC, CCG, CCC, CGG, CAG, CGC) are less abundant in IMODNs. The compositions of other trinucleotides are comparable in both IMODNs and non-IMODNs.

#### Tetranucleotide composition [TetNC]

In order to understand the role of tetranucleotides, we computed the composition of tetranucleotides in IMODNs and non-IMODNs. Similar to the above observations, the frequently occurring tetranucleotides in IMODNs contain thymine; viz., TGCC, CTGC, GCTG, ATGC, TGCT (Fig. S2 and Table S4). However, it is interesting to note that there are certain tetranucleotides that are preferred in IMODNs but do not contain thymine such as CCCC, GGGG and GCCC.

#### Pentanucleotide composition [PNC]

We also calculated and compared the pentanucleotide composition of IMODNs and non-IMODNs. We found that the pentanuceotides (e.g., CCCCC, GGGGG, TTTTT) containing only one kind of nucleotides were preferred in IMODNs. Although, stretches like GCCCC, TGCTG, CTGCC, ATGCC, GCTGC, GATGC and CTGCT are also frequent in IMODNs (Fig. S3 and Table S5).

### Identification of Motifs in IMODNs

In addition to the above compositional analysis, we also identified motifs and their occurrences in IMODNs and non-IMODNs. We discovered motifs using the Motif-EmeRging and with Classes-Identification (MERCI) program on the IMODN2193 dataset containing 2193 experimentally validated IMODNs and 2193 non-IMODNs. We found that out of 100 degenerate motifs, the top 20 motifs (Top20) provide sufficient coverage in the datasets. The motif “TCGTCG-T” was the most exclusive motif found in the immunomodulatory sequences of the dataset IMODN2193 with the coverage of 186 sequences. On the other hand, the most exclusive motif in non-immunomodulatory sequences was “AG-C-C-C-GCC-C” that covered 114 non-IMODNs (Table S9). We also discovered motifs in the sequences of the dataset IMODN966P that contains 966 palindromic IMODNs and an equal number of palindromic non-IMODNs. It was observed that the most prevalent motif in palindromic IMODNs is “TCGTCG” with the coverage of 143 sequences. In palindromic non-IMODN dataset, the prevailing motif was “G-GG-AG-G-GC-G” covering 142 non-IMODNs (Table S10). Similarly, we have also extracted the motifs with relaxed conditions for each dataset used in our study (Tables S9 and S10).

### Prediction of immunomodulatory oligonucleotide

The above compositional and motif analyses indicate that IMODNs and non-IMODNs differ in their nucleotide sequences. These features (e.g., sequence composition, motif occurrence) can be used to develop methods to discriminate between IMODNs and non-IMODNs. We developed support vector machine (SVM)-based models to predict IMODNs. These models were trained and tested on the IMODN2193_train dataset containing 1754 experimentally validated IMODNs and 1754 non-IMODNs.

#### Composition based models

As shown in the analysis section, the composition profiles of IMODNs and non-IMODNs (in the form of MNC, DNC, TNC, TetNC and PNC) have the potential to discriminate between IMODNs and non-IMODNs. We exploited these features to develop models on the dataset IMODN2193_train. The developed models using MNC, DNC and TNC achieved maximum Matthews Correlation Coefficient (MCC) of 0.52, 0.68 and 0.71 with accuracies of 76.0%, 84.24%, and 85.38% respectively. We also evaluated the performance of our models using threshold independent parameter and achieved the maximum Area Under the Curve (AUC) of 0.82, 0.92 and 0.93 for MNC, DNC and TNC respectively. Similarly, prediction models were developed using TetNC and PNC that achieved a maximum MCC of 0.72 and 0.75 with the AUC of 0.94 and 0.94 respectively (Table 1). It is clear that models developed using the pentanucleotide composition (PNC) perform better than the other composition-based models. We also developed and evaluated the SVM-based model using PNC on the realistic IMODN2193R_train dataset and achieved a maximum accuracy of 90.07% with MCC of 0.59 (Table S6).

#### Models based on hybrid features

In the hybrid approach, we combined the motif information with the PNC-based models. We observed a marginal improvement in the case of hybrid models developed using ‘exclusive motifs’ instead of motifs searched under relaxed parameters. We achieved the maximum performance of models developed on IMODN2193_train using the Top20 exclusive motifs and PNC (Table 2).

#### Prediction of palindromic immunomodulatory oligonucleotide

**Figure 3:**
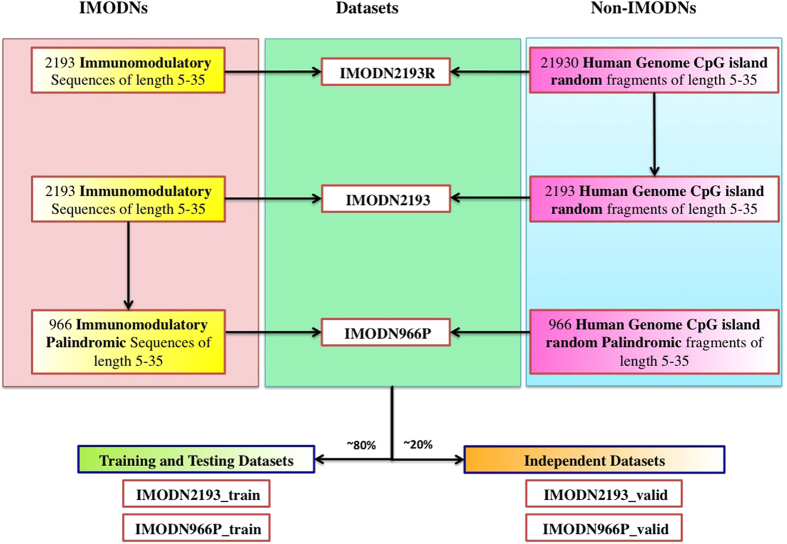
A representative scheme of datasets and their use in model development.

In addition to discriminate IMODNs from non-IMODNs, we have also developed models to predict palindromic IMODNs. These models were trained and tested on the IMODN966P_train dataset, containing 782 palindromic IMODNs and 782 non-IMODNs (Fig. 3). All models were evaluated using the five-fold cross-validation method. First, models were developed using various nucleotides compositions (e.g., MNC, TNC, PNC). These models achieved highest MCC of 0.81 with the accuracy of 90.35% in the case of PNC as the sequence feature (Table 1). Secondly, models were developed using the nucleotide composition and the Top20 exclusive IMODN motifs and achieved MCC of 0.84 (Table 2).

#### Performance of models on independent dataset

Although we evaluated the performance of our models using the five-fold cross-validation, the possibility of the performance biases cannot be ruled out. Thus, it is important to assess a model on an independent dataset (not used for training or testing) to measure the realistic performance of a model^14^. In this study, we evaluated the performance of the best models on the independent datasets. Our best IMODNs prediction model (developed using PNC and motif) achieved the MCC of 0.72 with 86.22% accuracy on the independent dataset of IMODN2193_valid. This dataset contains 439 experimentally validated IMODNs and an equal number of non-IMODNs. Similarly, we evaluated the performance of our best model developed to predict palindromic IMODNs on an independent dataset IMODN966P_valid that contains 184 experimentally validated palindromic IMODNs and non-IMODNs. Our palindromic IMODNs prediction model achieved MCC of 0.81 with 90.49% accuracy. In both the cases, the performances of models on independent datasets were comparable to those of the models evaluated using the five-fold cross-validation techniques.

### VaccineDA

The principal aim of this study was to develop a platform that can be used to design IMODN-based vaccine adjuvants. In order to provide a comprehensive service to the scientific community, we developed an *in silico* platform known as ‘VaccineDA’. Brief descriptions of the menus and their submenus integrated into VaccineDA are given below.

#### IMODN

This module allows users to predict whether their query oligonucleotide sequence is an IMODN or not. It also predicts palindromic IMODNs. This module allows a user to select a desired model to predict from a list of given models developed in this study. Users can also select a threshold to achieve desired coverage/sensitivity or specificity. Users can submit multiple oligonucleotides for prediction and this option is suitable for virtual screening.

#### DNASCAN

This menu performs a fixed-length window scan of the query sequence and predicts whether the window sequence would be a potential IMODN or not. In this way, it maps the IMODN regions of a query DNA sequence. This module is useful to those researchers who wish to discover regions/oligonucleotides in a natural DNA sequence.

#### DESIGN

This menu helps the user to mutate a query sequence in order to improve its immunomodulatory potential. It has two submenus, namely the QMSCAL (Quantitative Matrix Score Calculator) and the VIRTUAL SCREENING. QMSCAL allows users to identify the minimum mutations required of an oligonucleotide to change it from non-IMODNs to IMODNs or *vice versa* based on the quantitative matrix. In the case of VIRTUAL SCREENING, the server first generates analogs for a given oligonucleotide by making all possible mutations at each position. In the next step, it predicts IMODN potential of each analog to allow the user to select analogs of the desired immunomodulatory score.

#### MAPPING

This menu facilitates the identification of known IMODNs in a given sequence or the human genome based on the similarity search. The similarity search tool allows the user to perform a BLAST against experimentally validated IMODN sequences using the BLAST-SHORT. It is possible that a designed IMODN sequence is already present in the human genome, which is not desirable. The GENOME SCAN tool is designed to check given IMODNs sequences in the human genome. The DIGEST tool virtually digests a query sequence with the given restriction enzymes and identifies the potential IMODNs among the generated fragments.

#### MOTIF

This menu can be used to identify palindromes or exclusive motifs found in either IMODNs or non-IMODNs. The PALINDROME tool searches for palindromes in a query DNA sequence. Users can use the UNIQUE MOTIFS menu to search for motifs that are exclusive to IMODNs and/or non-IMODNs. The IMODN-specific motifs, if present in the query sequence, will render immunomodulatory properties to the sequence.

## Discussion

It has long been observed that an individual exposed to cowpox gained natural long-term protection against smallpox^23^. This accidental observation of natural long-term protection against a pathogen led to the development of vaccines. Our immune system not only has the ability to fight against a pathogen, but also provides long-term protection against that pathogen using memory cells (B- or T-cells)^24^. This knowledge led to the development of traditional vaccines, where the killed pathogen is exposed to the immune system of a patient^24^. These traditional vaccines have limitations that include toxicity due to unwanted pathogen components, and which is not required for the protective effect^25^.

Over the years, our knowledge about the immune system has improved remarkably and led to the development of subunit vaccines, where the immune system is stimulated by the exposure to an antigen or protein instead of the whole pathogen to stimulate the immune system. One of the major limitations of subunit (antigen or epitope)-based vaccines is that they may activate the adaptive immunity but not the innate immunity. Thus, these subunit vaccines require effective adjuvants to stimulate the immune system by activating the innate immune system^20^. In the past, a number of attempts have been made to develop effective adjuvants using the empirical approaches. Some of these adjuvants have been approved for clinical use^26^ like alum (an aluminium salt), AS04 (a TLR4 ligand), emulsions (MF59), etc. The rational design of adjuvants is replacing the empirical approaches as our understanding of the innate immune system is increasing with time^20^.

In this study, we proposed the development of models to design ODN-based adjuvants. These ODNs had a high abundance of CpG islands that are recognized by the TLR9 receptor. In the past, it was shown that the stimulation of TLR9 by oligonucleotides is sequence-dependent^27^. In this study, we collected and compiled experimentally validated IMODNs from the literature that can activate our innate immunity. The compositional analysis of the immunomodulatory sequences revealed the motifs having the maximum difference between the compositions of the positive (IMODNs) and the negative (non-IMODNs) sequences. The motifs found in IMODNs are rich in thymine (T) (e.g., TT, TTT, GTT, TTG, TTC, TTTTT, etc.). Such an observation is consistent with a previous report that the IMODNs, rich in thymine along with CpG dinucleotides, are stronger inducers of the immune response^28^. Further, motif analysis using the MERCI program revealed the preferential occurrence of thymine-rich motifs in IMODNs. Using these compositional features and the MERCI motifs, we developed SVM-based models that were able to predict immunomodulatory ODNs with accuracies ranging from ~76% to ~91%.

## Conclusion

In the present study, the first ever attempt has been made to develop an *in silico* platform to design ODN-based vaccine adjuvants. Most of these ODNs are CpG containing sequences that can stimulate the innate system. In this study, we used only sequence-based features to predict immunomodulatory ODNs with reasonable accuracy. We did not consider other features such as backbone modifications in ODNs. It has been shown recently that sugar-phosphate backbone and chemical modification of the backbone affects the immunomodulatory activity of an ODN^29^,^30^. We hope that in the future studies, researchers will consider all these factors to develop better models. In addition, our models were developed on highly redundant sequences (unique sequence, may have up to 99% similarities in few sequences) because of the limited data on IMODNs. In the future, models should be developed on non-redundant data. Despite these limitations, we have created a powerful web-based resource, ‘VaccineDA’, for the rational design of vaccine adjuvants. As described above, VaccineDA has numerous *in silico* modules to provide various facilities to the users required to aid in the design of ODN-based vaccine adjuvants. We hope that the scientific community will be highly benefited from our new web-based service available freely at http://crdd.osdd.net/raghava/vaccineda/.

## Methods

### Datasets

In this study, we examined 75 patents related to oligonucleotide-based vaccine adjuvants and collected 7945 IMODNs. After removing identical sequences, there were 2473 unique IMODNs. 2193 experimentally validated IMODNs having nucleotides between 5 to 35 were obtained after removing very small and long sequences. Unfortunately, we were unable to collect experimentally validated non-IMODNs from the literature. Thus, we randomly obtained oligonucleotides from the human genome and assigned them non-IMODNs (with an assumption that the human DNA is non-immunomodulatory). Overall the process of creating different types of datasets used in this study is shown in Fig. 3.

We obtained oligonucleotides having 5 to 35 nucleotides, which were generated by random fragmentation of the CpG islands in the human genome (GRCh37/hg19) obtained from the UCSC database^31^. We assigned these randomly generated oligonucleotides as non-IMODNs. We created two datasets namely IMODN2193, a balanced dataset and IMODN2193R, a realistic dataset. Our balanced dataset IMODN2193 contains 2193 experimentally validated IMODNs and an equal number of non-IMODNs (randomly generated CpG islands from the human genome). Similarly, the realistic dataset IMODN2193R contains experimentally identified 2193 IMODNs and 21930 non-IMODNs (10 times to IMODNs). In addition, we also created a palindromic dataset IMODN966P that included 966 palindromic IMODNs (palindromic motifs induce IFN-α^22^) and an equal number of palindromic non-IMODNs. In order to develop models, we created training and validation datasets from the above datasets.

#### Training datasets

In order to train our models, we generated IMODN2193_train dataset that contains 80% of oligonucleotide sequences in the IMODN2193 dataset; 1754 IMODNs and an equal number of non-IMODNs. Similarly, we created the IMODN2193R_train dataset that contains 80% of the oligonucleotide sequences in the IMODN2193R dataset. In order to train our models to predict palindromic IMODNs, we generated the IMODN966P_train dataset from the IMODN966P that included 782 palindromic IMODNs and an equal number of palindromic non-IMODNs.

#### Independent or validation dataset

It is important to validate a model on an independent dataset not used for training or testing the model. Thus, we created validation or independent datasets that contain the remaining 20% oligonucleotide sequences not included in the above training datasets. The dataset IMODN2193_valid includes 439 IMODNs and 439 non-IMODNs that are 20% of sequences in the IMODN2193. Similarly, we generated IMODN966P_valid from the IMODN966P that contains 184 IMODNs and an equal number of non-IMODNs.

In order to compare the composition of IMODNs and non-IMODNs with the composition of the genomes, we computed the composition of human and bacterial genomes. In the case of the bacterial genomes, we downloaded the genome sequences of 11 of the most dangerous human pandemic bacteria^32^ from UCSC, namely *Bordetella pertussis*, *Corynebacterium diphtheriae*, *Mycobacterium leprae*, *Mycobacterium tuberculosis*, *Rickettsia prowazekii*, *Salmonella enterica*, *Shigella dysenteriae*, *Streptococcus pyogenes*, *Treponema pallidum*, *Vibrio cholera* and *Yersinia pestis*.

### Compositional analysis

We computed different compositional aspects of oligonucleotides such as mono-, di-, tri-, tetra- and pentanucleotide compositions. These features have already been used for analysis and prediction of different classes of nucleotides^33^. These mononucleotide, dinucleotide, trinucleotide, tetranucleotide and pentanucleotide compositions are represented by vectors of dimension 4, 16, 64, 256 and 1024 respectively. For the visualization, the compositions of oligonucleotides were presented as Circos plots, generated using the web-based Circos tool^34^.

### Motif-based analysis

The motif identification is essential for immunomodulatory sequences. In the current study, we used the MERCI program to extract significant motifs in IMODNs and non-IMODNs. The MERCI program^35^ extracts significant motifs by comparing positive (IMODNs in this study) and negative (non-IMODNs in this study) sequences. We extracted 100 degenerate motifs in following four categories: 1) exclusive motifs (motifs present exclusively in positive or negative sequences); 2) Relaxed_200 (these motifs may be shared in positive and negative dataset at the maximum of 200 sequences); 3) Relaxed_300 (these motifs may occur in positive and negative dataset with maximum sequences of 300); 4) Relaxed_400 (here the sharing of motifs could be up to 400 IMODNs). Out of 100 motifs extracted in each category, “Top10” and “Top20” motifs were extracted based on their exclusive sequence coverage.

### Hybrid models

In the hybrid approach, we incorporated the motif information into the SVM-based model. We assigned a score of ‘+1’ for oligonucleotide having immunomodulatory motifs and ‘−1’ for oligonucleotides having a motif present in non-immunomodulatory sequences. This motif-based weight assignment was combined with SVM score obtained using the SVM-based model.

### Quantitative Matrix (QM)

In the literature, Quantitative Matrix (QM) has been used to evaluate toxic peptides^36^ and cytokine stimulating peptides^37^, etc. The QM was generated on the basis of the probability or frequency of a nucleotide at a particular position. The QM score represents the independent contribution of a particular nucleotide in IMODN and non-IMODNs. Single letter code indicates the four nucleotides, and nucleotide positions range from 1 to 35. Since our datasets had a maximum length of 35 nucleotides, so our QM is represented by a dimension of 4 × 35 matrix (Table S7). We also created QMs for dinucleotide motifs where contribution of all possible dinucleotide is computed for each position. In the case of dinucleotide-based QM, it is presented by a matrix of dimension 16 × 34 (Table S8).

### Support Vector Machine

In this study, models were developed using SVM, which has been exploited heavily to develop prediction models^33^. We optimized different kernels and parameters using the freely available program SVM^Light^ (Support Vector Machine)^38^ to select the best performing models on different datasets.

## Additional Information

**How to cite this article**: Nagpal, G. *et al.* VaccineDA: Prediction, design and genome-wide screening of oligodeoxynucleotide-based vaccine adjuvants. *Sci. Rep.* **5**, 12478; doi: 10.1038/srep12478 (2015).

## Supplementary Material

Supplementary Figures

Supplementary Information

## Figures and Tables

**Table 1:** The performance of models developed on training datasets-IMODN2193_train and IMODN966P_train using various compositional features.

Feature	IMODN2193_train						IMODN966P_train					
	Thres	Sen	Spec	Acc	MCC	AUC	Thres	Sen	Spec	Acc	MCC	AUC
MNC	0.2	76.17	75.83	76.00	0.52	0.82	0.1	78.39	78.26	78.32	0.57	0.86
DNC	−0.1	83.64	84.83	84.24	0.68	0.92	−0.1	84.53	83.63	84.08	0.68	0.92
TNC	−0.1	85.12	85.63	85.38	0.71	0.93	−0.1	87.08	87.08	87.08	0.74	0.95
TetNC	−0.1	85.52	86.49	86.00	0.72	0.94	−0.1	89.64	89.90	89.77	0.80	0.96
PNC	−0.2	87.51	87.63	87.57	0.75	0.94	−0.2	90.15	90.54	90.35	0.81	0.97

**Thres** Threshold, **Sen** Sensitivity (%), **Spec** Specificity (%), **Acc** Accuracy (%), **MCC** Matthews Correlation Coefficient, **AUC** Area Under the Curve, **MNC** Monoucleotide Composition, **DNC** Dinucleotide Composition, **TNC** Trinucleotide Composition, **TetNC** Tetranucleotide Composition, **PNC** Pentanucleotide Composition.

**Table 2:** The performance of models developed using hybrid features on training datasets-IMODN2193_train and IMODN966P_train.

Feature	Motifs	Thres	IMODN2193_train					IMODN966P_train				
			Sen	Spec	Acc	MCC	AUC	Sen	Spec	Acc	MCC	AUC
Exclusive	Top10	−0.2	87.8	88.65	88.23	0.76	0.95	91.18	91.05	91.11	0.82	0.97
Exclusive	Top20	−0.2	88.03	89.17	88.6	0.77	0.96	91.18	92.71	91.94	0.84	0.98
Relax_200	Top10	−0.2	85.69	86.83	86.26	0.73	0.93	90.15	91.05	90.6	0.81	0.98
Relax_200	Top20	−0.2	86.09	86.83	86.46	0.73	0.93	90.41	91.05	90.73	0.81	0.97
Relax_300	Top10	−0.2	85.35	86.32	85.83	0.72	0.93	89.90	91.43	90.66	0.81	0.97
Relax_300	Top20	−0.2	85.92	86.89	86.4	0.73	0.93	90.15	91.05	90.6	0.81	0.97
Relax_400	Top10	−0.2	87.23	86.32	86.77	0.74	0.93	90.03	90.92	90.47	0.81	0.97
Relax_400	Top20	−0.2	87.17	87.00	87.09	0.74	0.94	90.15	90.66	90.41	0.81	0.97

**Exclusive** MERCI motifs found exclusively in positive sequences, **Top10** top 10 MERCI motifs in the category, **Relax_200** MERCI motifs found in positive sequences and up to 200 sequences in negative dataset.
