# Supplementary material for: VaccineDA: Prediction, design and genome-wide screening of oligodeoxynucleotide-based vaccine adjuvants
Source: Sci Rep. 2015 Jul 27;5:12478. doi: 10.1038/srep12478 (PMC4515643; doi:10.1038/srep12478)
Supplement: Supplementary Figures [file srep12478-s1.doc]

**VaccineDA : Prediction, design and genome-wide screening of oligodeoxynucleotide-based vaccine adjuvants**


Gandharva Nagpal^#^, Sudheer Gupta^#^, Kumardeep Chaudhary^#^, Sandeep Kumar Dhanda^#^, Satya Prakash, and Gajendra P. S. Raghava*


# Authors contributed equally
* Corresponding Author
Address: Bioinformatics Centre, CSIR-Institute of Microbial Technology, Chandigarh-160036, INDIA.
For correspondence please email: raghava@imtech.res.in

**Supplementary Figures**

**Figure S1**: Circos Plot of the average composition of trinucleotide motifs in IMODNs and non-IMODNs. The motifs are top 10 in terms of the difference between average composition in IMODNs and non-IMODNs. The width of the ribbons emerging from each trinucleotide and extending to IMODNs or non-IMODNs gives a comparative view of abundance.


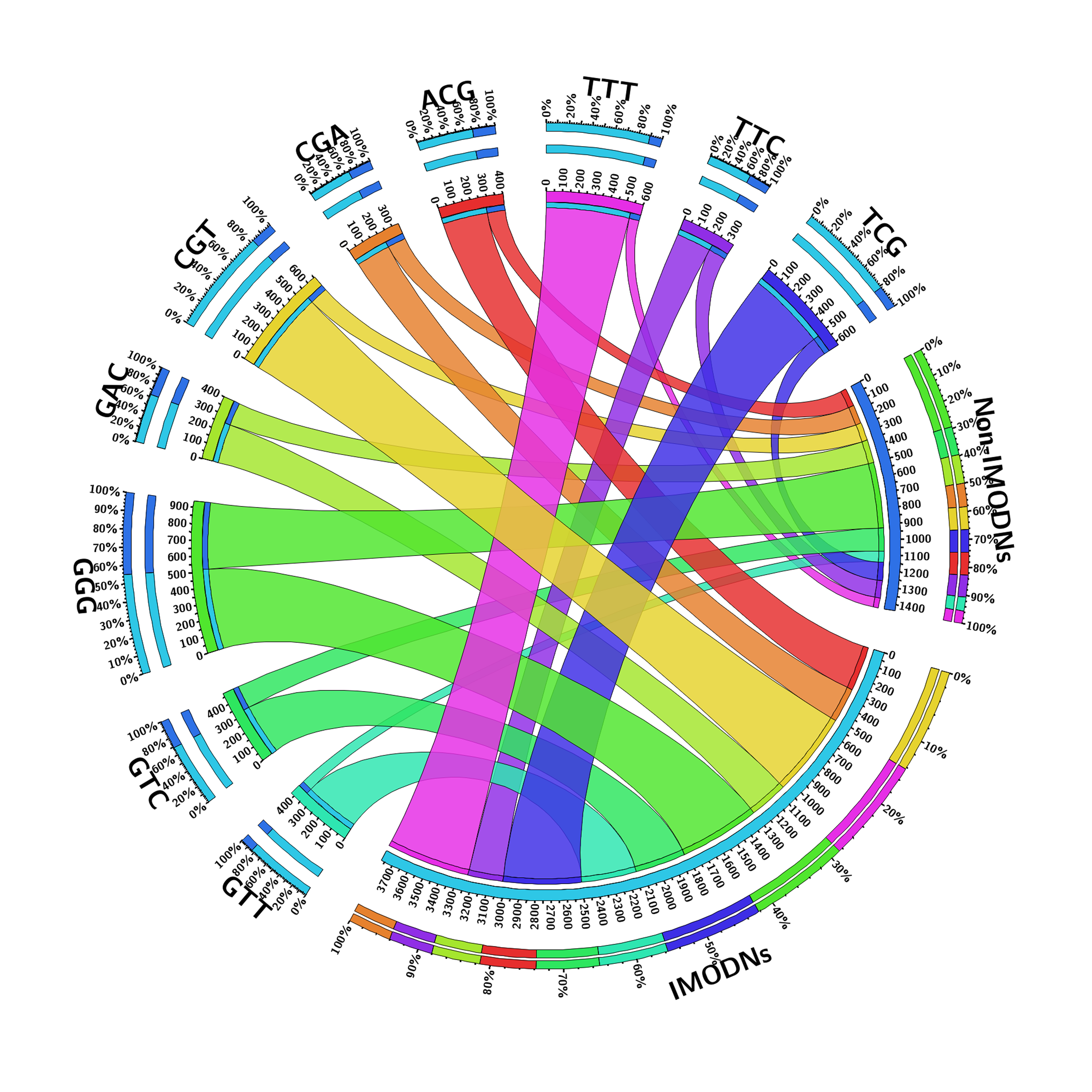


**Figure S2**: Circos Plot of the average composition of tetranucleotide motifs in IMODNs and non-IMODNs. The motifs are top 10 in terms of the difference between average composition in IMODNs and non-IMODNs. The width of the ribbons emerging from each tetranucleotide and extending to IMODNs or non-IMODNs gives a comparative view of abundance.


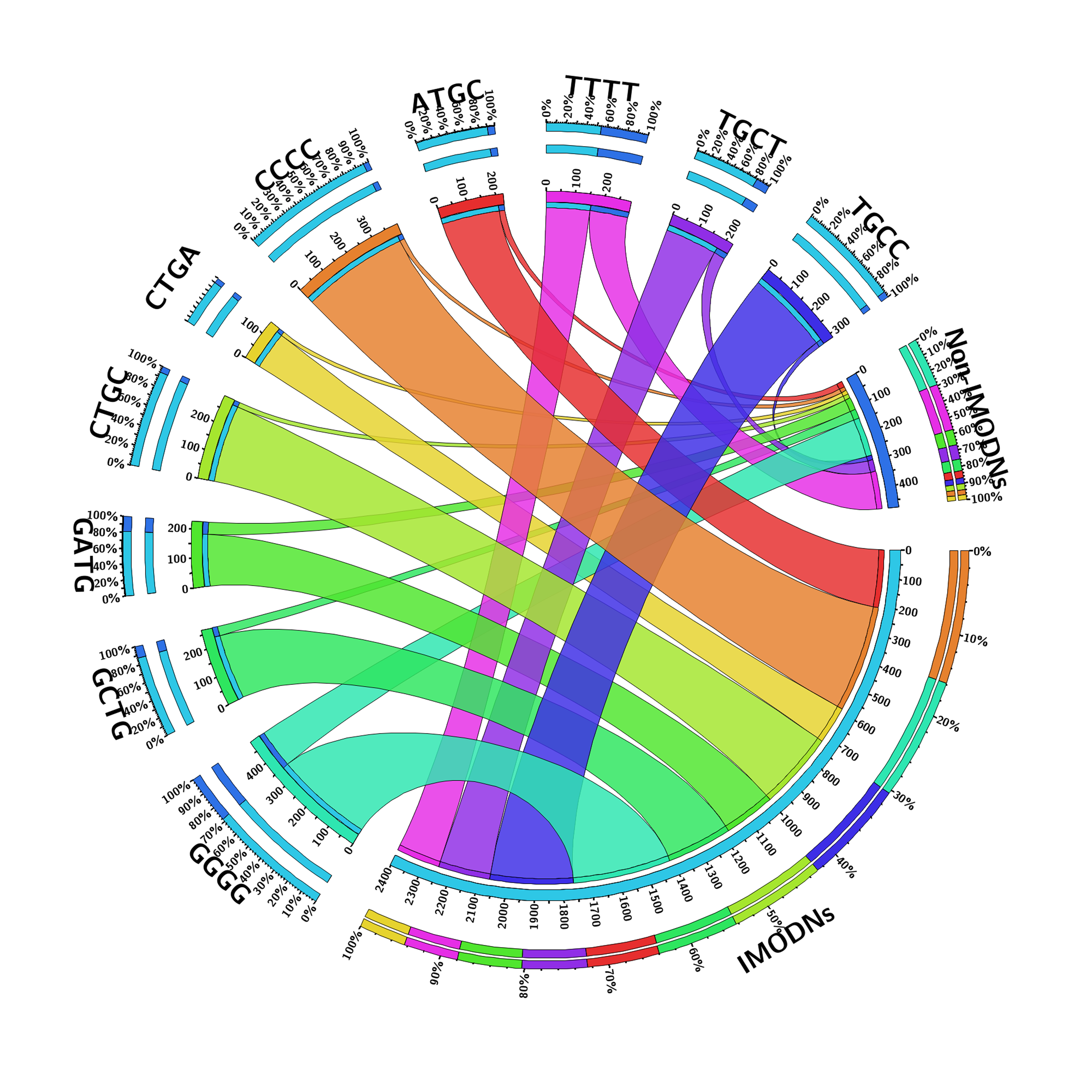


**Figure S3**: Circos Plot of the average composition of pentanucleotide motifs in IMODNs and non-IMODNs. The motifs are top 10 in terms of the difference between average composition in IMODNs and non-IMODNs. The width of the ribbons emerging from each pentanucleotide and extending to IMODNs or non-IMODNs gives a comparative view of abundance.

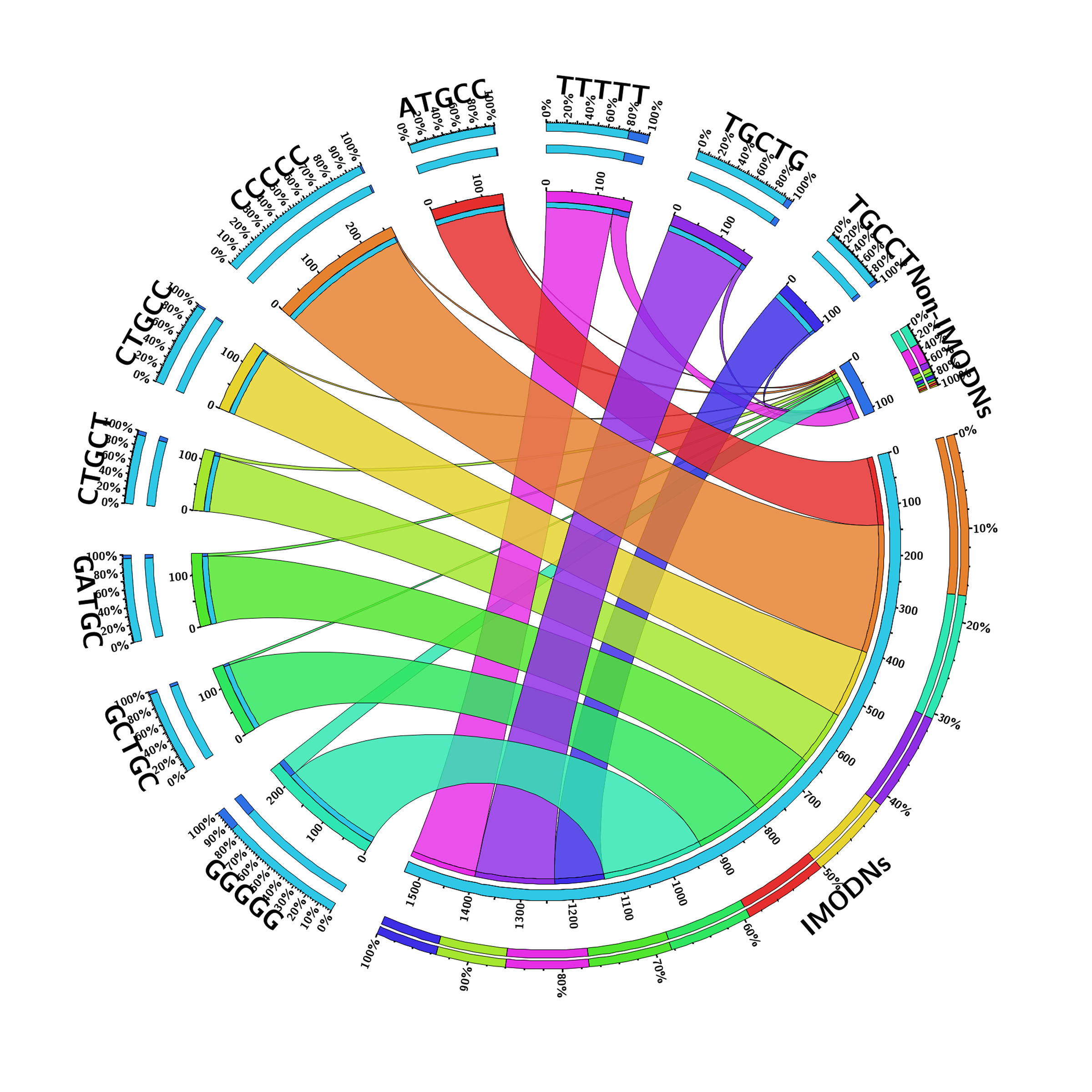


**Supplementary Table Legends**
**Table S1**: Percentage nucleotide composition (averaged) of IMODN and non-IMODN sequences in the dataset IMODN2193R.
**Table S2**: Percentage dinucleotide composition (averaged) of IMODN and non-IMODN sequences in the dataset IMODN2193R.
**Table S3**: Percentage trinucleotide composition (averaged) of IMODN and non-IMODN sequences in the dataset IMODN2193R.
**Table S4**: Percentage tetranucleotide composition (averaged) of IMODN and non-IMODN sequences in the dataset IMODN2193R.
**Table S5**: Percentage pentanucleotide composition (averaged) of IMODN and non-IMODN sequences in the dataset IMODN2193R.
**Table S6**: Performances of realistic dataset IMODN2193R; pentanucleotide composition model and hybrid models based on pentanucleotide composition and motif information of IMODNs.
**Table S7**: Position based quantitative matrix built from dataset IMODN2193 showing mononucleotide preference at all positions in the oligonucleotide sequences.
**Table S8**: Position based quantitative matrix built from dataset IMODN2193 showing dinucleotide preference at all positions in the oligonucleotide sequences.
**Table S9**: Preferred motifs found in main dataset (IMODN2193: positive immunomodulatory sequences and non-immunomodulatory negative sequences from human CpG islands) using MERCI program.
**Table S10**: Preferred motifs found in palindromic dataset derived from main dataset (IMODN966P: positive immunomodulatory sequences and non-immunomodulatory negative sequences from human CpG islands) using MERCI program.
